# Localization and Expression of Hsp27 and αB-Crystallin in Rat Primary Myocardial Cells during Heat Stress *In Vitro*

**DOI:** 10.1371/journal.pone.0069066

**Published:** 2013-07-19

**Authors:** Shu Tang, Rehana Buriro, Zhijun Liu, Miao Zhang, Islam Ali, Abdelnasir Adam, Jörg Hartung, Endong Bao

**Affiliations:** 1 College of Veterinary Medicine, Nanjing Agricultural University, Nanjing, China; 2 College of Animal Science and Technology, Jinling Institute of Technology, Nanjing, China; 3 Institute for Animal Hygiene, Animal Welfare and Farm Animal Behaviour, University of Veterinary Medicine Hannover, Foundation, Hannover, Germany; Center for Cancer Research, National Cancer Institute, United States of America

## Abstract

Neonatal rat primary myocardial cells were subjected to heat stress *in vitro*, as a model for investigating the distribution and expression of Hsp27 and αB-crystallin. After exposure to heat stress at 42°C for different durations, the activities of enzymes expressed during cell damage increased in the supernatant of the heat-stressed myocardial cells from 10 min, and the pathological lesions were characterized by karyopyknosis and acute degeneration. Thus, cell damage was induced at the onset of heat stress. Immunofluorescence analysis showed stronger positive signals for both Hsp27 and αB-crystallin from 10 min to 240 min of exposure compared to the control cells. According to the Western blotting results, during the 480 min of heat stress, no significant variation was found in Hsp27 and αB-crystallin expression; however, significant differences were found in the induction of their corresponding mRNAs. The expression of these small heat shock proteins (sHsps) was probably delayed or overtaxed due to the rapid consumption of sHsps in myocardial cells at the onset of heat stress. Our findings indicate that Hsp27 and αB-crystallin do play a role in the response of cardiac cells to heat stress, but the details of their function remain to be investigated.

## Introduction

Exposure to high temperature without proper cooling could be dangerous and even fatal, as high temperatures can damage the brain and other internal organs; sudden exposure to heat is reported to lead to heart attack, stroke and cardiac arrest [1], [2]. Heat stroke is defined as an increase in body temperature to higher than 40.6°C due to environmental heat exposure and lack of thermoregulation [2]. Several factors influence the induction of heat stress, such as medication and age-related physiological changes, which can predispose individuals to heat stroke [3].

Hyperthermia can induce cellular damage and apoptosis in many different cell types [4], [5]. In particular, pathological changes in heart cells in response to stress have been investigated in transported pigs [6] as well as chickens [7]. For instance, a 60% death rate related to heart failure caused by transport stress was reported for pigs in Great Britain [8].

It has been reported that the expression of heat shock proteins (Hsps) is increased when cells are exposed to elevated temperatures or other stresses, such as disease [9]. Hsps are a class of functionally related proteins that play regulatory roles in preventing stress injury, protein synthesis (folding and unfolding) and degradation, immune response generation and apoptosis [10]–[13], and they are found in virtually all living organisms, from bacteria to humans. When cells are exposed to high temperature, Hsps act as molecular chaperones that counteract the formation of aberrantly folded polypeptides and allow their correct refolding during stress recovery [14], [15]. Hsps are named according to their molecular weight, such as Hsp90 (molecular weight = 90 kD), Hsp60, Hsp70 and Hsp27 [14].

Small heat shock proteins (sHsps) are characterized by low molecular masses (12–43 kDa) and a conserved C-terminal domain [16]. sHsps are found in most organisms, where they are induced in response to stress and are involved in protecting cells from various unfavorable conditions such as anoxia, UV, glucose deprivation, toxic radicals and carcinogens [17]–[19]. sHsps were first detected in the heart, where they were found to be mainly distributed in the cytosol of cardiac cells, but were also present in the perinuclear region and nucleus [20]–[23]. In humans, 10 sHsps have been characterized, but only a few of them, such as Hsp27, Hsp22 and αB-crystallin, are true Hsps that display enhanced synthesis in response to several stresses [24], [25].

Hsp27 is considered to be a potential diagnostic marker for cancer [26]. Notably, small oligomers formed by Hsp27 can sustain cytoskeleton integrity [27], [28], which is required to maintain normal muscular function. αB-Crystallin occurs as large aggregates, comprising two types of related subunits (A and B). It is expressed widely in many tissues and plays a role in the pathogenesis of several diseases [29]. αB-crystallin is reported to be overexpressed under ischemia and reperfusion conditions *in vitro* where it has cytoprotective effects [30], [31]. αB-Crystallin accounts for up to 3% of the total protein in the heart; moreover, the overexpression of αB-crystallin in cultured cardiac myocytes protects them from ischemia-induced cell death and stabilizes microtubules [32].

Hsp27 and αB-crystallin are found in many cell types, especially smooth muscle cells. Some researchers have confirmed that Hsp27 and αB-crystallin are abundantly produced in response to various types of stress in cardiac and skeletal muscles as well as in the brain *in vivo* [29], [33]. Although Hsp27 and αB-crystallin display a wide range of cellular properties, they are mostly known for their ability to interact with cytoskeletal proteins and play the role of chaperones. However, the function of Hsp27 and αB-crystallin in heat-stressed myocardial cells *in vitro* is still unknown.

Heat-related deaths are sometimes difficult to investigate as it is difficult to measure environmental and body core temperatures during field investigations [34], [35]. Moreover, it is difficult to systematically investigate the mechanisms of stress-induced cell damage and alterations in cellular metabolism *in vivo* because of the presence of numerous confounding environmental variables. Therefore, in the present study, we have conducted an *in vitro* examination of variations in the expression and localization of Hsp27 and αB-crystallin in primary myocardial cell models in which heat shock was induced after exposure to different durations of heat stress at 42°C.

## Materials and Methods

### Cell Culture and Heat Stress Treatment

Two-day-old rat primary myocardial cells were provided by Shanghai Fu Meng Biological Technology Ltd. Cells were cultured for 72 h until the fusion rate was higher than 90%. The cultured cells were divided into nine groups: control group (0 min) and eight experimental groups exposed to heat stress for 10 min, 20 min, 40 min, 60 min, 120 min, 240 min, 360 min and 480 min. The cells were exposed to heat stress as rapidly as possible by quickly transferring them from a 37°C incubator to a 42°C humidified atmosphere containing 5% CO_2_ and 95% air.

### Enzymatic Activities in the Supernatant of Cultured Myocardial Cells

The supernatant media from myocardial cell cultures were collected from both the heat stress and control groups, and stored at −80°C. The activity of aspartate aminotransferase (AST, C010), lactate dehydrogenase (LDH, A-020-1), and creatine kinase (CK, A032) in all the media samples was measured according to the instructions given in the commercial kits (Nanjing Jiancheng Biochemical Reagent Co., Nanjing, China). Each measurement was repeated five times. The enzyme activities were formulated by the enzyme unit (U), which represents the amount of a particular enzyme.

### Cytopathological Examination

Myocardial cells (3–5×10^5^ cells grown on glass coverslips) were heat stressed at 42°C, fixed in 95% ethanol for 10 min at room temperature (RT), stained with hematoxylin and eosin (H&E), as described previously [36] and examined by light microscopy.

### Immunofluorescence Analysis

Myocardial cells (2–8×10^4^ cells in 35-mm^2^ plates) were fixed directly on dishes using pre-cooled 3% formaldehyde in phosphate buffer solution (PBS) for 30 min at room temperature (RT), and permeabilized with 0.1% Triton X-100 in PBS for 15 min. After blocking with 5% skim milk in PBS for 1 h, a 1∶200 dilution of each, anti-rat Hsp27 monoclonal antibody (ab78307, Abcam, USA) and αB-crystallin monoclonal antibody (ab13496, Abcam), was added to the coverslips and they were incubated in a moist chamber for 1 h at 37°C. After washing with PBS three times, the coverslips were incubated with rhodamine red-conjugated goat anti-mouse IgG antibody at a 1∶100 dilution (BA1089, Boster, China) at 37°C for 1 h. After washing with PBS again, the coverslips were dyed with DAPI solution (H-1000, Vector, USA). Myocardial cells were observed using an immunofluorescence microscope (Cx41-32rfl, Olympus, Japan).

### Western Blotting Analysis

Myocardial cells were harvested directly in 2× Laemmli buffer [37]. The protein content was measured using the BCA protein assay kit (Pierce). Samples were boiled for 5 min. Equal amounts of protein (20 µg) were loaded on a 10% acrylamide:bisacrylamide (30∶0.8) gel. After separation, the proteins were transferred onto a nitrocellulose membrane by electrotransfer (200 mA, 2 h). The membrane was blocked with 5% non-fat milk in Tris-buffered saline (20 mM Tris-HCl (pH 7.6) and 137 mM NaCl) containing 0.1% Tween-20 (TBST) for 1 h at RT. Then, the membrane was incubated with anti-rat Hsp27 monoclonal antibody (ab78307, Abcam, Japan) and αB-crystallin monoclonal antibody (ab13496, Abcam, USA) at a 1∶1000 dilution and anti-rat β-actin monoclonal antibody (ab8224, Abcam, USA) at a 1∶1000 dilution for 16 h at 4°C. After the membrane was washed with TBST, it was further incubated with peroxidase-conjugated goat anti-mouse IgG antibody (BA1038, Boster, China) at a 1∶1000 dilution at RT for 1 h. The antibody-antigen complexes were detected using western blotting luminal reagent. The bands on the developed film were quantified with Quantity One 4.6.2 software (Bio-Rad, USA). The density of each band was normalized to that of β-actin protein.

### Detection of hsp27 and αB-Crystallin mRNA by Fluorescence Quantitative Real-time PCR

#### Isolation of total RNA and reverse transcription

After myocardial cells were exposed to heat stress at 42°C, they were washed with PBS, and total RNA was isolated using the TRIzol reagent (15596-026, Invitrogen, USA) according to the manufacturer’s instructions. The concentration of RNA was determined by a spectrophotometer (Mx3000P, USA) at 260 nm. Serial dilutions of RNA were prepared with ribonuclease-free water, and 2 µg of each sample was synthesized into DNA using the Transcript M-MLV kit (28025013, Gibco, USA) following the manufacturer’s protocol. Reaction products were stored at −80°C.

#### Design of PCR primers

Primer sets were specifically designed to anneal to each target mRNA. The sequences of *hsp27, αB-Crystallin* and *β-actin* mRNA were obtained from the National Center for Biotechnology Information’s (NCBI) Genbank (accession nos. BC081945.1 and NM_031144.2, respectively). The primers were designed using the Primer Premier 5.0 software for conventional and RT-PCR amplification. Primer sequences for the genes were: CGTGGTGGAGATCACTGGCAAGC and CGGGCCTCGAAAGTGACCGG for *hsp27*, and CACGAAGAGCGCCAGGACGA and CGTCGGCTGGGATCCGGTACT for *αB-crystallin.* The expected size of the *hsp27* and *αB-crystallin* PCR product was 216 bp and 153 bp respectively. The expected size of the *β-actin* PCR product was 161 bp, and the primer sequences were TGCTCCTCCTGAGCGCAAGT and ACGCAGCTCAGTAACAGTCCGC.

#### Quantitative real-time PCR

Each DNA sample (2 µL, 25 times dilution) was suspended in 2× SYBR Premix Ex Taq (DRR041S, Takara, China) with primers (25 pmol of sense and antisense primers) and double-distilled water to make up a total volume of 25 µL. Quantitative PCR was performed using an ABI 7300 qPCR thermocycler (7300, Applied Biosystems, USA). The thermal profile was established according to the manufacturer’s protocol. Briefly, enzyme activation was carried out at 95°C for 3 min followed by 40 cycles of denaturation at 95°C for 5 s and annealing and elongation at 53°C for 30 s. For each run, a negative control tube without DNA was analyzed along with the experimental samples. A 2-fold dilution series of the template was used in the quantitative PCR (qPCR) reactions. The levels of *hsp27* mRNA and α*B-crystallin* mRNA were normalized to those of β-actin using the following formula:

Relative quantity of hsp27/αB-crystallin mRNA = 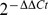


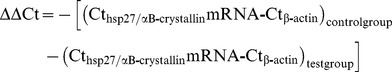



### Statistical Analysis

Differences between the heat stress groups and the control group were analyzed by one-way analysis of variance (ANOVA) followed by the LSD multiple comparison test, using the Statistical Package for Social Sciences (SPSS version 20.0 for Windows). Results were expressed as the mean ± SD of at least three independent experiments. *P*-values that were <0.05 were considered to indicate statistical significance, unless otherwise indicated. All experiments were performed in triplicate (n = 3).

## Results

### Cardiomyocyte Damage-related Enzyme Activities in the Supernatant of Myocardial Cells

**Figure 1:**
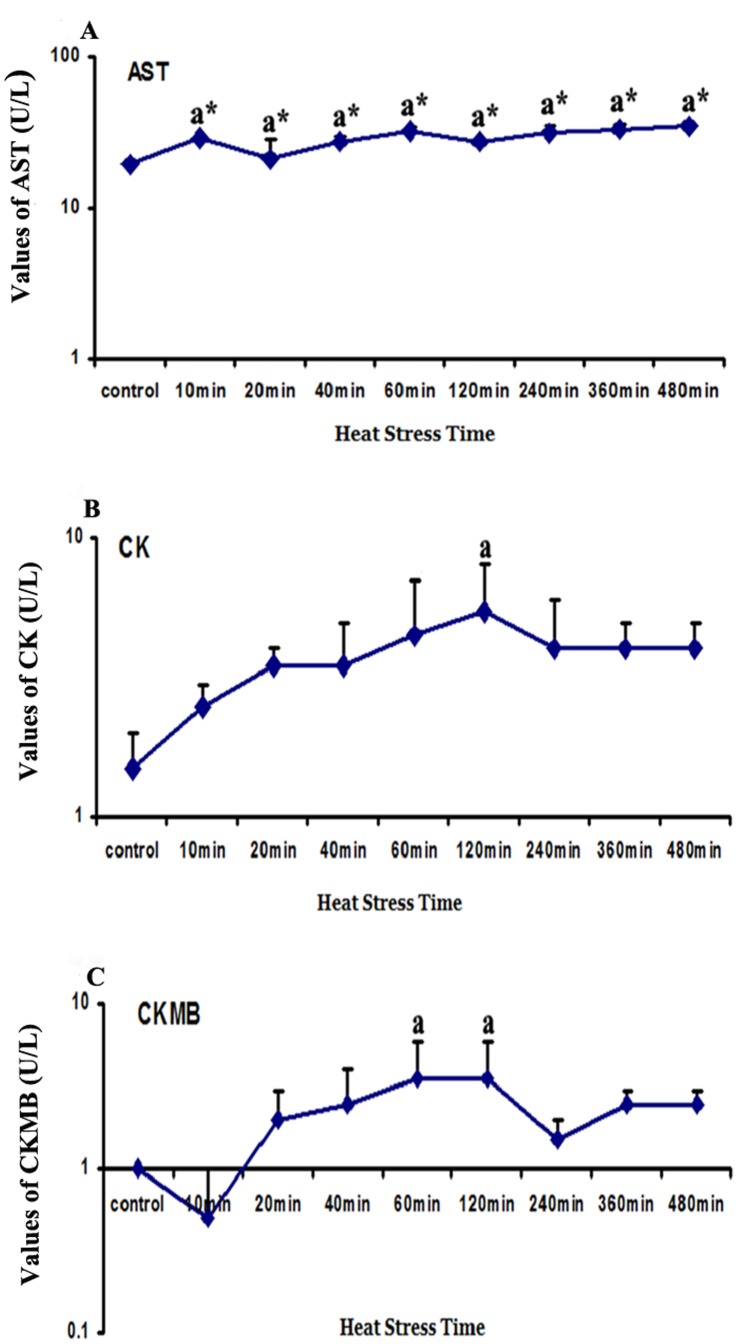
Time course of enzyme activities in the supernatant of myocardial cells exposed to heat stress. A: The activity levels of AST showed an obvious increase from 10 min of heat exposure at 42°C; B: The activity levels of CK showed an increasing tendency and decreased after 120 min of heat stress; C: The activity levels of CK-MB showed an increasing tendency, reached peak levels after 60 min and 120 min of exposure to heat stress, and then decreased after 120 min. ^a^ *P*<0.05; ^a*^ *P*<0.01.

The AST, CK, and CKMB (creatine kinase-MB) levels in the media of primary myocardial cell cultures are shown in Figure 1. The AST activities in the media of the heat-stressed groups were significantly higher (*P*<0.01) in comparison with the control group. The level of CK activity displayed an induction tendency (*P*>0.05) and increased significantly at 120 min of heat stress (*P*<0.05). The levels of CKMB were increased significantly (*P*<0.05) at 60 and 120 min of heat stress. Although the enzyme levels of CK and CKMB started to decrease gradually after 240 min of heat stress, they were still higher than those of the control group.

### Cytopathological Changes in Primary Myocardial Cells after Heat Stress *in vitro*

**Figure 2:**
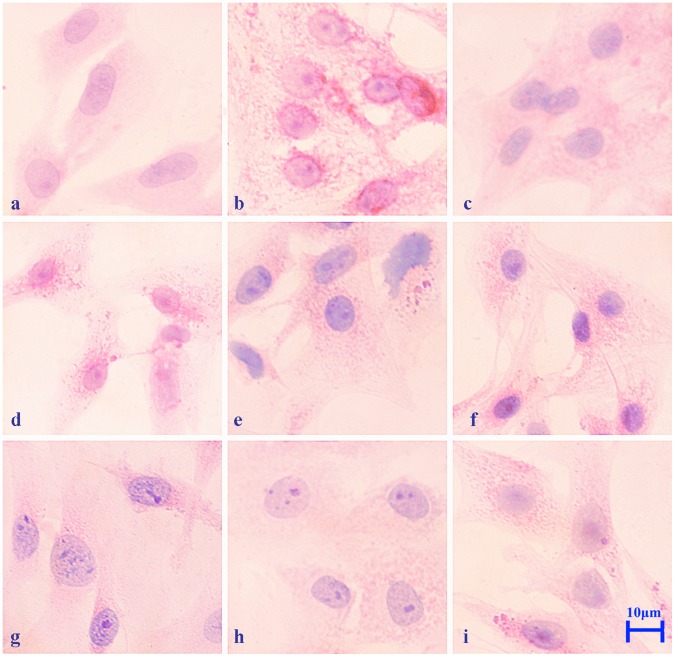
Pathological changes in primary rat myocardial cells exposed to heat stress *in vitro* at 42°C. Myocardial cells were incubated at 37°C, heat stressed at 42°C for 10, 20, 40, 60, 120, 240, 360 and 480 min, stained with H&E and photographed using a Carl Zeiss optical microscope equipped with an imaging system (400×). Scale bar = 10 µm. a. Control cells (37°C). b. After 10 min of heat stress, acute granular degeneration was observed in the cytoplasm compared to control cells. c. After 20 min of heat stress, the cytoplasm of the swelling myocardial cells was obviously cloudy. d. After 40 min, numerous red granules were observed in the cytoplasm. e. After 60 min of heat stress, the nuclei were observed to be markedly basophilic and the cell sizes were enlarged. f. After 120 min of heat stress, karyopyknosis was observed. g. After 240 min, markedly basophilic nuclei and intracellular vacuoles were observed in the heat stressed myocardial cells. h. After 360 min, the number of intracellular granules in the cytoplasm of swollen myocardial cells decreased. i. After 480 min of heat stress, the cells remained enlarged and degeneration was evident compared with the control group.

The cytopathological changes that occurred in primary rat myocardial cells after they were heat stressed at 42°C *in vitro* are shown in Figure 2. Acute degeneration characterized by numerous fine granular particles in the cytoplasm and enlarged cell size were observed from the beginning (10 min) to the end of heat stress (480 min). However, from 40 min to 240 min of heat stress, the stress damage characterized by the obvious formation of karyopyknosis and intracellular granules were identified. After 240 min of heat stress, the main lesions of the stressed myocardial cells were cytomorphosis (enlarged size) and acute degeneration. There was no obvious pathological change in the control group.

### Immunofluorescence Findings for Hsp27 and αB-crystallin Expression in Myocardial Cells after Heat Stress

**Figure 3:**
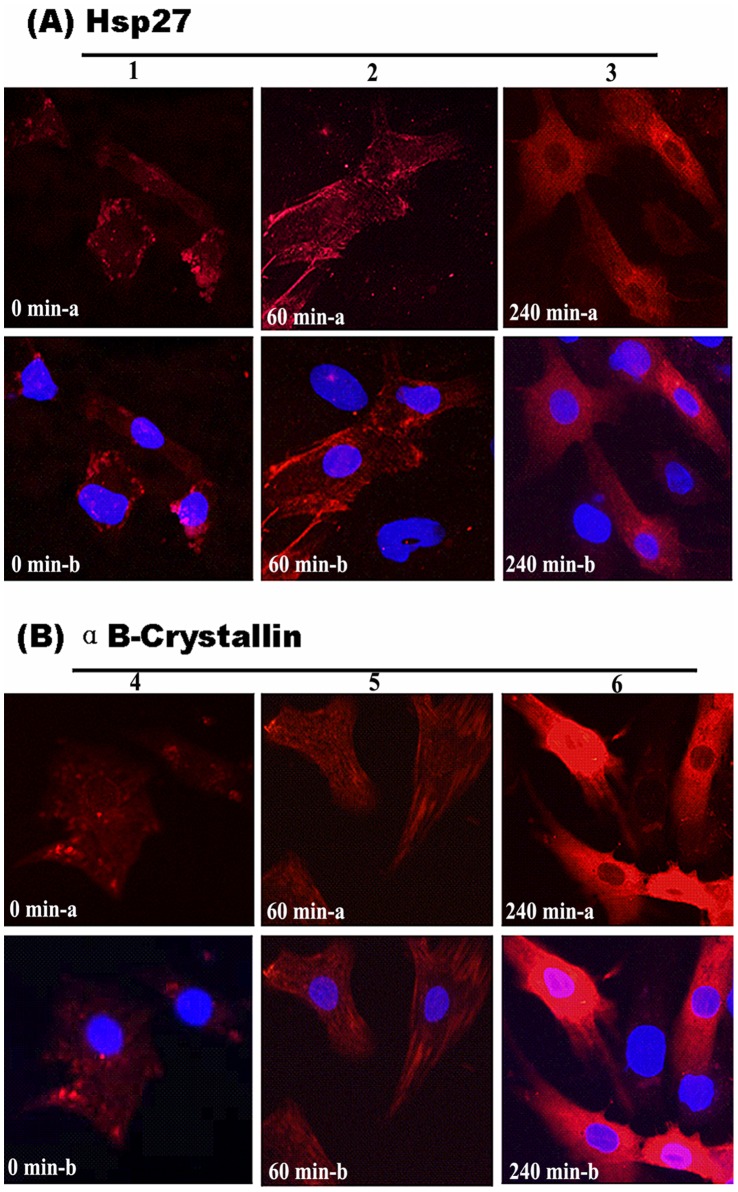
Distribution of Hsp27 and αB-crystallin in control and heat-stressed myocardial cells. A: Hsp27, a: Hsp27 (Rhodamine); b: merge. B: αB-Crystallin, a: αB-Crystallin (Rhodamine ); b: merged. 1: Smeared and granular Hsp27 signals can be seen distributed in the cytoplasm and nucleus of the control myocardial cells; 2: Stronger positive signals for Hsp27 were observed in both the nucleus and cytoplasm at 60 min of heat stress compared with the control group; 3: Hsp27 was distributed mainly in the cytoplasm after 240 min of heat stress, but weak signals were observed in the nucleus. 4: αB-Crystallin was found distributed in the form of smashed granules in the cytoplasm of the control myocardial cells. 5: Stronger αB-crystallin signals were found scattered in the cytoplasm of myocardial cells exposed to heat stress compared with the control group after 60 min of heat stress. 6: After 240 min of heat stress, the strongest positive signals for αB-crystallin were observed in both the cytoplasm and nucleus of myocardial cells.

Distribution and localization of Hsp27 and αB-crystallin both in control and the heat-stressed groups are shown in Figure 3A and 3B, respectively.

Although Hsp27 was distributed in both the nucleus and the cytoplasm of myocardial cells after stress induction, stronger signals were found in the cytoplasm. In the unstressed state, Hsp27 signals were distributed as smears, but they were weakly distributed in the cytoplasm as long as very little can be found in the nuclei of myocardial cells. After 60 min of heat stress, Hsp27 signals distributed in both the nucleus and cytoplasm of myocardial cells were obviously observed compared with the control group. However, Hsp27 was mainly distributed in the cytoplasm, and weak signals were observed in the nucleus of myocardial cells after heat treatment for 240 min.

αB-Crystallin signals were characterized by tiny smashed granules mixed with larger stained granules scattered in the cytoplasm of myocardial cells in the control group. αB-Crystallin signals were obviously and consistently present in the cytoplasm of myocardial cells both in the heat-stressed groups and the control group. After 60 min of exposure to heat stress, αB-crystallin signals were found scattered in the cytoplasm. The strongest positive signals for αB-crystallin were found scattered in both the nucleus and cytoplasm of myocardial cells at 240 min of heat stress.

### Variation in the Expression of Hsp27 and αB-crystallin in Myocardial Cells after Heat Stress

**Figure 4:**
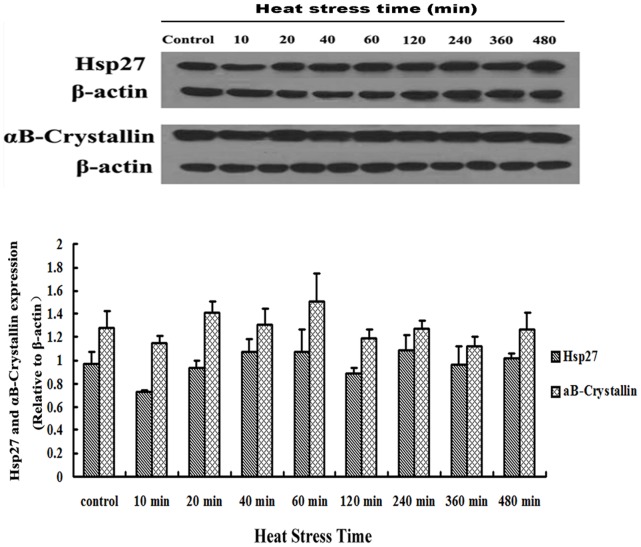
Hsp27 and αB-crystallin protein expression in myocardial cells after heat stress. Western blotting results (normalized to β-actin protein) showed that during the 480 min of heat stress exposure, the expression of Hsp27 did not show significant variations (*P*>0.05) compared to the control group. αB-Crystallin expression also did not show any obvious variation during the 480 min of heat shock.

Variations in the expression of Hsp27 and αB-crystallin, normalized to myocardial cell β-actin levels, are displayed in Figure 4. Although there were differences in their expression, it was not significant (*P*>0.05) compared to the control group during 480 min of heat shock exposure *in vitro*.

### Variations in hsp27 mRNA and αB-crystallin mRNA Transcription in Myocardial Cells after Heat Stress

**Figure 5:**
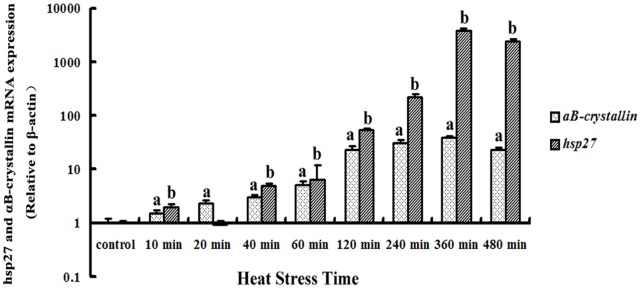
Transcription levels of *hsp27* mRNA and *αB-crystallin mRNA* in myocardial cells after heat stress. qPCR Ct values for hsp27 and αB-crystallin were normalized against β-actin. Expression of this housekeeping gene did not change in response to heat stress. The results showed that the transcription levels of hsp27 mRNA increased immediately and significantly (*P*<0.01) from 10 min after the onset of heat shock until 480 min, peaking at 360 min. The transcription levels of *αB-crystallin* mRNA also showed a significant (*P*<0.01) increase from 10 min after exposure to heat stress until 480 min, and peaked at 360 min. For *αB-crystallin* mRNA, ^a^ *P*<0.05; ^a*^, *P*<0.01. For *hsp27* mRNA, ^b^ *P*<0.01.

The transcription levels of *hsp27* mRNA and *αB-crystallin* mRNA, normalized to myocardial cell *β-actin* mRNA, are displayed in Figure 5. Ct values for hsp27 and αB-crystallin were normalized against β-actin. The expression of this housekeeping gene did not change in response to heat stress. The transcription levels of *hsp27* mRNA were increased immediately and significantly (*P*<0.01) after exposure to heat shock. With increase in the amount of exposure time, the transcription levels of *hsp27* mRNA persistently increased. The transcription level of *hsp27* mRNA was eight-fold higher at 120 min than that at 60 min of heat stress, and reached peak levels at 360 min, at which point it was 18-fold higher than that at 240 min. The transcription levels of *αB-crystallin* mRNA in the myocardial cells showed a significant (*P*<0.01) increase as soon as they were exposed to high temperature (at 10 min). After 120 min of heat exposure, the transcription level of *αB-crystallin* mRNA in the heat-stressed myocardial cells was four-fold higher, and it reached the highest level at 360 min of exposure.

## Discussion

We have found that Hsp27 and αB-crystallin show changes in their localization and expression in rat primary myocardial cells exposed to heat stress, based on the results of western blotting, immunofluorescence analysis, and qPCR.

We first confirmed the induction of cellular damage by determining the levels of the mitochondrial enzymes CK, CK-MB, LDH and AST, which are used as cardiac function indicators to investigate damage to the heart not only in humans but also in livestock [37]–[43]. In the present study, these enzymes were useful and provided for rapid identification of damage to the heart at the beginning of heat treatment. CK, as well as its isozyme, plays a particularly important role in tissues with large and fluctuating energy demands such as those in the muscle and brain [44]. CK is one of the most abundant enzymes found in heart cells, and it is used as a specific diagnostic factor for cardiac damage [45]. When the membrane permeability of myocardial cells increases or these cells are damaged due to external stress, CK is released from myocardial cells and its levels are therefore increased in cell media or blood serum [44]. AST is mainly distributed in the heart, brain, liver, muscles, kidneys and other organs, which can also be regarded as key elements for the diagnosis of heart cell damage [46]. The cytopathological changes of the heat-stressed primary myocardial cells, characterized by nuclear shrinkage (karyopyknosis) and acute granular degeneration, occurred *in vitro* as early as 10 min exposure to heat stress, and were particularly evident from 60 min to 240 min. Our results suggest that myocardial cells are considerably damaged even after 10 min of heat stress at 42°C, and also that the level of AST can be considered an early indicator of cardiac damage.

With regard to the role of sHSPs in cardiac damage, our immunofluorescence results indicate that the distribution densities of Hsp27 and αB-crystallin in the myocardial cells increased with time of exposure to heat stress and varied from that in the control cells even after exposure to heat for a short time. The results are in agreement with another study, according to which these proteins were expressed dynamically in rat primary myocardial cells when they were subjected to stress [47]. According to another report, Hsp27 was mainly distributed in the cytoplasm, and heat stress increased the phosphorylation of Hsp27, which led to its transfer to the nucleus in PC12 cells [48]. In our study, we found that although Hsp27 and αB-crystallin were distributed both in the nucleus and cytoplasm of myocardial cells of the control and heat stress groups, stronger signals were found in the cytoplasm with heat stress, especially after 240 min of exposure. This may be related to their role in the refolding of denatured proteins induced by heat exposure. Also, αB-crystallin showed stronger signals after 240 min of heat stress as compared to Hsp27. This is probably related to its role as a skeletal protein which can stabilize myofilament proteins through selective interactions with actin, titin, nebulette and the intermediate filaments, desmin and vimentin [49].

Our Western blotting results showed that there was no significant increase in the expression of both Hsp27 and αB-crystallin during 480 min of heat stress. In contrast, the transcription of both *hsp27* mRNA and *αB-crystallin* mRNA showed a dramatic and similar increase from the onset of heat stress (according to the results of qPCR). This suggests that sHsp expression was delayed or overtaxed due to rapid consumption of both sHsps at the onset of heat stress, since they are required for myocardial cell balance in response to stress [6]. It has been reported that overexpression of mutant αB-crystallin (R120G) in the heart can lead to cardiomyocyte death, dilation, and heart failure [50], suggesting that even molecular chaperones like Hsps need to be in a relatively stable state to play a protective role *in vivo* and *in vitro.*

Similar to our results, it has been shown that *hsp27* mRNA and *αB-crystallin* mRNA are overexpressed in heart and other smooth muscles after heat stress, but the expression of their corresponding proteins does not differ [29]. However, after the addition of myogenic factor, Hsp27 and αB-crystallin expression increases significantly [29]. This is probably linked to the function of these Hsps in the protection of cells against stress or to a specific function in a particular tissue [6], [24]. Hsp27 and αB-crystallin may modulate interaction between cellular factors by forming small or large oligomers, undergoing phosphorylation, and inducing cell–cell contact [51]. However, the detailed functions of these two sHsps in myocardial cells are not fully understood.

Thus, our results together indicate that Hsp27 and αB-crystallin do indeed play a regulatory role in the response of cardiac cells to heat stress, but the details of the mechanism still need to be investigated.

## References

[1] Bettaieb A, Averill-Bates DA (2008) Thermotolerance induced at a fever temperature of 40 degrees C protects cells against hyperthermia-induced apoptosis mediated by death receptor signalling. Biochem Cell Biol 86: 521–538. 19088800 10.1139/O08-136

[2] Herbst J, Gilbert JD, Byard RW (2011) Urinary incontinence, hyperthermia, and sudden death. J Forensic Sci 56: 1062–1063. 21470226 10.1111/j.1556-4029.2011.01760.x

[3] Sinha AK, Ghacha R, Youmbissi JT, Khursany IA (2001) Classic heat stroke in a case of simple hypohydrosis with “bad prognostic indicators” but a remarkable recovery. Ren Fail 23: 727–730. 11725920 10.1081/jdi-100107370

[4] Hildebrandt B, Wust P, Ahlers O, Dieing A, Sreenivasa G, et al (2002) The cellular and molecular basis of hyperthermia. Crit Rev Oncol Hematol 43: 33–56. 12098606 10.1016/s1040-8428(01)00179-2

[5] Lepock JR (2004) Role of nuclear protein denaturation and aggregation in thermal radiosensitization. Int J Hyperthermia 20: 115–130. 15195506 10.1080/02656730310001637334

[6] Zhang M, Xin L, Bao E, Hartung J, Yue Z (2011) Variation in the expression of Hsp27, alphaB-crystallin mRNA and protein in heart and liver of pigs exposed to different transport times. Res Vet Sci 90: 432–438. 20659751 10.1016/j.rvsc.2010.06.028

[7] Yu J, Bao E, Yan J, Lei L (2008) Expression and localization of Hsps in the heart and blood vessel of heat-stressed broilers. Cell Stress Chaperones 13: 327–335. 18350374 10.1007/s12192-008-0031-7 PMC2673943

[8] Sains A (1980) Deaths in transit: what British surveys show. Pig Farming 28: 40–41.

[9] De Maio A (1999) Heat shock proteins: facts, thoughts, and dreams. Shock 11: 1–12. 10.1097/00024382-199901000-00001 9921710

[10] Bukau B, Horwich AL (1998) The Hsp70 and Hsp60 chaperone machines. Cell 92: 351–366. 9476895 10.1016/s0092-8674(00)80928-9

[11] Lindquist S (1986) The heat-shock response. Annu Rev Biochem 55: 1151–1191. 2427013 10.1146/annurev.bi.55.070186.005443

[12] Mehlen P, Kretz-Remy C, Preville X, Arrigo AP (1996) Human hsp27, Drosophila hsp27 and human alphaB-crystallin expression-mediated increase in glutathione is essential for the protective activity of these proteins against TNFalpha-induced cell death. EMBO J 15: 2695–2706. 8654367 PMC450205

[13] Polla BS, Kantengwa S, Gleich GJ, Kondo M, Reimert CM, et al (1993) Spontaneous heat shock protein synthesis by alveolar macrophages in interstitial lung disease associated with phagocytosis of eosinophils. Eur Respir J 6: 483–488. 8491297

[14] Li Z, Srivastava P (2004) Heat-shock proteins. Curr Protoc Immunol Appendix 1: Appendix 1T. 10.1002/0471142735.ima01ts58 18432918

[15] Santoro MG (2000) Heat shock factors and the control of the stress response. Biochem Pharmacol 59: 55–63. 10605935 10.1016/s0006-2952(99)00299-3

[16] Kargul J, Laurent GJ (2012) Small heat shock proteins: molecular protectors against the disease. Int J Biochem Cell Biol 44: 1587. 22743309 10.1016/j.biocel.2012.06.022

[17] Acunzo J, Katsogiannou M, Rocchi P (2012) Small heat shock proteins HSP27 (HspB1), alphaB-crystallin (HspB5) and HSP22 (HspB8) as regulators of cell death. Int J Biochem Cell Biol 44: 1622–1631. 22521623 10.1016/j.biocel.2012.04.002

[18] Haslbeck M, Franzmann T, Weinfurtner D, Buchner J (2005) Some like it hot: the structure and function of small heat-shock proteins. Nat Struct Mol Biol 12: 842–846. 16205709 10.1038/nsmb993

[19] Van Montfort R, Slingsby C, Vierling E (2001) Structure and function of the small heat shock protein/alpha-crystallin family of molecular chaperones. Adv Protein Chem 59: 105–156. 11868270 10.1016/s0065-3233(01)59004-x

[20] Bhat SP, Nagineni CN (1989) alpha B subunit of lens-specific protein alpha-crystallin is present in other ocular and non-ocular tissues. Biochem Biophys Res Commun 158: 319–325. 2912453 10.1016/s0006-291x(89)80215-3

[21] Gernold M, Knauf U, Gaestel M, Stahl J, Kloetzel PM (1993) Development and tissue-specific distribution of mouse small heat shock protein hsp25. Dev Genet 14: 103–111. 8482014 10.1002/dvg.1020140204

[22] Haslbeck M (2002) sHsps and their role in the chaperone network. Cell Mol Life Sci 59: 1649–1657. 12475175 10.1007/PL00012492 PMC11337447

[23] Iwaki T, Kume-Iwaki A, Liem RK, Goldman JE (1989) Alpha B-crystallin is expressed in non-lenticular tissues and accumulates in Alexander’s disease brain. Cell 57: 71–78. 2539261 10.1016/0092-8674(89)90173-6

[24] Kim KK, Kim R, Kim SH (1998) Crystal structure of a small heat-shock protein. Nature 394: 595–599. 9707123 10.1038/29106

[25] Wettstein G, Bellaye PS, Micheau O, Bonniaud P (2012) Small heat shock proteins and the cytoskeleton: an essential interplay for cell integrity? Int J Biochem Cell Biol 44: 1680–1686. 22683760 10.1016/j.biocel.2012.05.024

[26] Taba K, Kuramitsu Y, Ryozawa S, Yoshida K, Tanaka T, et al (2010) Heat-shock protein 27 is phosphorylated in gemcitabine-resistant pancreatic cancer cells. Anticancer Res 30: 2539–2543. 20682980

[27] Huot J, Houle F, Spitz DR, Landry J (1996) HSP27 phosphorylation-mediated resistance against actin fragmentation and cell death induced by oxidative stress. Cancer Res 56: 273–279. 8542580

[28] Lavoie JN, Lambert H, Hickey E, Weber LA, Landry J (1995) Modulation of cellular thermoresistance and actin filament stability accompanies phosphorylation-induced changes in the oligomeric structure of heat shock protein 27. Mol Cell Biol 15: 505–516. 7799959 10.1128/mcb.15.1.505 PMC232001

[29] Sugiyama Y, Suzuki A, Kishikawa M, Akutsu R, Hirose T, et al (2000) Muscle develops a specific form of small heat shock protein complex composed of MKBP/HSPB2 and HSPB3 during myogenic differentiation. J Biol Chem 275: 1095–1104. 10625651 10.1074/jbc.275.2.1095

[30] Bluhm WF, Martin JL, Mestril R, Dillmann WH (1998) Specific heat shock proteins protect microtubules during simulated ischemia in cardiac myocytes. Am J Physiol 275: H2243–2249. 9843825 10.1152/ajpheart.1998.275.6.H2243

[31] Ray PS, Martin JL, Swanson EA, Otani H, Dillmann WH, et al (2001) Transgene overexpression of alphaB crystallin confers simultaneous protection against cardiomyocyte apoptosis and necrosis during myocardial ischemia and reperfusion. FASEB J 15: 393–402. 11156955 10.1096/fj.00-0199com

[32] Garrido C, Paul C, Seigneuric R, Kampinga HH (2012) The small heat shock proteins family: the long forgotten chaperones. Int J Biochem Cell Biol 44: 1588–1592. 22449631 10.1016/j.biocel.2012.02.022

[33] Kourtis N, Nikoletopoulou V, Tavernarakis N (2012) Small heat-shock proteins protect from heat-stroke-associated neurodegeneration. Nature 490: 213–218. 22972192 10.1038/nature11417

[34] Thomes P, Rajendran M, Pasanban B, Rengasamy R (2010) Cardioprotective activity of Cladosiphon okamuranus fucoidan against isoproterenol induced myocardial infarction in rats. Phytomedicine 18: 52–57. 20638259 10.1016/j.phymed.2010.06.006

[35] Yu RJ, Wang ZY, Fang JB, Xu YC, Zhu XJ (2006) Analysis of relationship between injury and disease by cardiac functional indicators in hypertension rat after trauma. Fa Yi Xue Za Zhi 22: 21–23. 16524179

[36] Zhu L, Bao E, Zhao R, Hartung J (2009) Expression of heat shock protein 60 in the tissues of transported piglets. Cell Stress Chaperones 14: 61–69. 18548335 10.1007/s12192-008-0055-z PMC2673904

[37] Bao E, Sultan KR, Nowak B, Hartung J (2008) Expression and distribution of heat shock proteins in the heart of transported pigs. Cell Stress Chaperones 13: 459–466. 18465207 10.1007/s12192-008-0042-4 PMC2673930

[38] Buyukokuroglu ME, Taysi S, Buyukavci M, Bakan E (2004) Prevention of acute adriamycin cardiotoxicity by dantrolene in rats. Hum Exp Toxicol 23: 251–256. 15222403 10.1191/0960327104ht443oa

[39] Georgopoulos C, Welch WJ (1993) Role of the major heat shock proteins as molecular chaperones. Annu Rev Cell Biol 9: 601–634. 8280473 10.1146/annurev.cb.09.110193.003125

[40] Lin YH, Chiu JH, Tung HH, Tsou MT, Lui WY, et al (2001) Preconditioning somatothermal stimulation on right seventh intercostal nerve territory increases hepatic heat shock protein 70 and protects the liver from ischemia-reperfusion injury in rats. J Surg Res 99: 328–334. 11469906 10.1006/jsre.2001.6177

[41] Mitchell MA, Sandercock DA (1995) Creatine kinase isoenzyme profiles in the plasma of the domestic fowl (Gallus domesticus): effects of acute heat stress. Res Vet Sci 59: 30–34. 8525081 10.1016/0034-5288(95)90026-8

[42] Saravanan G, Ponmurugan P, Sathiyavathi M, Vadivukkarasi S, Sengottuvelu S (2011) Cardioprotective activity of Amaranthus viridis Linn: Effect on serum marker enzymes, cardiac troponin and antioxidant system in experimental myocardial infarcted rats. Int J Cardiol 165: 494–498. 21962802 10.1016/j.ijcard.2011.09.005

[43] Zhang M, Yue Z, Liu Z, Islam A, Rehana B, et al (2012) Hsp70 and HSF-1 expression is altered in the tissues of pigs transported for various periods of times. J Vet Sci 13: 253–259. 23000582 10.4142/jvs.2012.13.3.253 PMC3467400

[44] Schlattner U, Tokarska-Schlattner M, Wallimann T (2006) Mitochondrial creatine kinase in human health and disease. Biochim Biophys Acta 1762: 164–180. 16236486 10.1016/j.bbadis.2005.09.004

[45] Hekimsoy Z, Oktem IK (2005) Serum creatine kinase levels in overt and subclinical hypothyroidism. Endocr Res 31: 171–175. 16392619 10.1080/07435800500371706

[46] Oswald GA, Smith CC, Betteridge DJ, Yudkin JS (1986) Determinants and importance of stress hyperglycaemia in non-diabetic patients with myocardial infarction. Br Med J (Clin Res Ed) 293: 917–922. 10.1136/bmj.293.6552.917 PMC1341710 3094714

[47] Geum D, Son GH, Kim K (2002) Phosphorylation-dependent cellular localization and thermoprotective role of heat shock protein 25 in hippocampal progenitor cells. J Biol Chem 277: 19913–19921. 11912188 10.1074/jbc.M104396200

[48] Mearow KM, Dodge ME, Rahimtula M, Yegappan C (2002) Stress-mediated signaling in PC12 cells - the role of the small heat shock protein, Hsp27, and Akt in protecting cells from heat stress and nerve growth factor withdrawal. J Neurochem 83: 452–462. 12423255 10.1046/j.1471-4159.2002.01151.x

[49] Christians ES, Ishiwata T, Benjamin IJ (2012) Small heat shock proteins in redox metabolism: implications for cardiovascular diseases. Int J Biochem Cell Biol 44: 1632–1645. 22710345 10.1016/j.biocel.2012.06.006 PMC3412898

[50] Maloyan A, Sanbe A, Osinska H, Westfall M, Robinson D, et al (2005) Mitochondrial dysfunction and apoptosis underlie the pathogenic process in alpha-B-crystallin desmin-related cardiomyopathy. Circulation 112: 3451–3461. 16316967 10.1161/CIRCULATIONAHA.105.572552 PMC1398051

[51] Parcellier A, Schmitt E, Brunet M, Hammann A, Solary E, et al (2005) Small heat shock proteins HSP27 and alphaB-crystallin: cytoprotective and oncogenic functions. Antioxid Redox Signal 7: 404–413. 15706087 10.1089/ars.2005.7.404

